# Supplementary material for: Investigation of the Frying Fume Composition During Deep Frying of Tempeh Using GC-MS and PTR-MS
Source: Molecules. 2024 Oct 25;29(21):5046. doi: 10.3390/molecules29215046 (PMC11547179; doi:10.3390/molecules29215046)
Supplement: Supplementary file 1 [file molecules-29-05046-s001.zip › molecules-3256596-supplementary.pdf]

Supplementary Materials

Investigation of the frying fume composition during deep frying of Tempeh using GC-MS and PTR-MS

Rohmah Nur Fathimah[1], Tomasz Majchrzak[1,*]

1 Department of Analytical Chemistry, Faculty of Chemistry, Gdańsk University of Technology, 80-233 Gdańsk, Poland
* Correspondence: tomasz.majchrzak@pg.edu.pl

S1. Oil quality parameters

Table S1. TPM value and thermal stability parameter of fresh and fried oils

Frying Condition	TPM (%)		Rancimat (h)	
	Rapeseed Oil	Palm Oil	Rapeseed Oil	Palm Oil
Fresh Oil	5.50	10.67	12.53	15.57
180 °C - 1st	5.83	10.50	9.23	15.32
180 °C - 2nd	6.50	11.17	7.01	12.12
180 °C - 3rd	7.83	11.00	6.47	11.83
180 °C - 4th	8.67	11.17	6.72	5.80
180 °C - 5th	9.33	13.83	5.57	1.77
160 °C	5.17	9.83	6.52	15.78

S2. Fragmentation pattern

Table S2. Fragmentation pattern for hexanal

Compound	Ion (*m/z*)				
Hexanal	43	47	55	83	101 (MH^+)
Signal (cps)	1550	20220	1599	12420	2949
%	4%	52%	4%	32%	8%

*An experiment was done on a single-component mixture of approximately 1 g/L hexanal in methanol. The vial was heated to 60 °C for 30 min and then placed directly in front of the PTR-MS transfer line; E/N = 100 Td. The ratio of the registered ions may differ from those obtained in the real-time monitoring scenario described in the manuscript.

S3. Impact of repeated use cycles, temperature and type of oil on VOCs emission profile

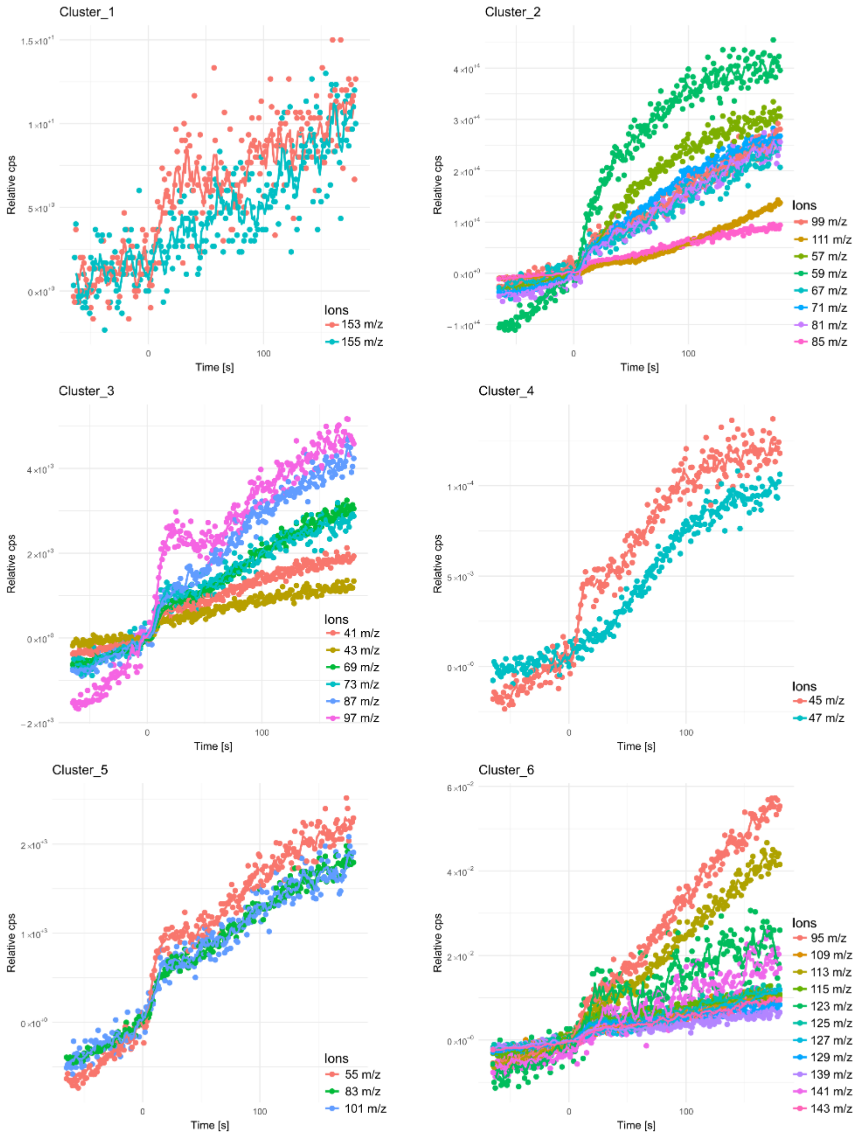

Figure S1. The emission profiles of individual VOCs (grouped in clusters) monitored during deep frying of tempeh in rapeseed oil; frying at 180 °C, fresh oil; y-axis – relative counts per second, where zero is the start of frying; x-axis – frying time before frying (below zero) and after placing tempeh; the line is the result of smoothing with moving average with step = 5.

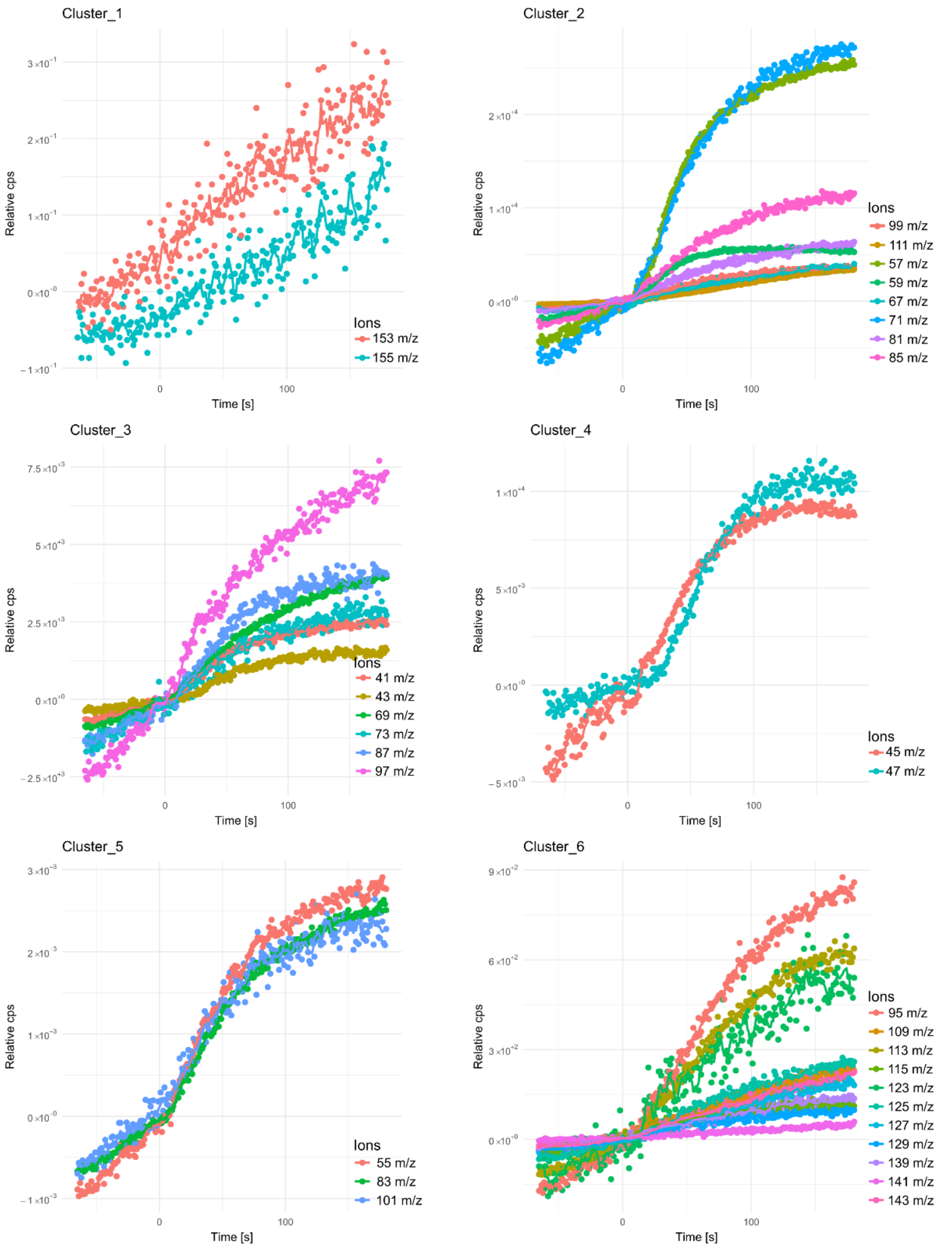

Figure S2. The emission profiles of individual VOCs (grouped in clusters) monitored during deep frying of tempeh in rapeseed oil; frying at 180 °C, second use of oil; y-axis – relative counts per second, where zero is the start of frying; x-axis – frying time before frying (below zero) and after placing tempeh; the line is the result of smoothing with moving average with step = 5

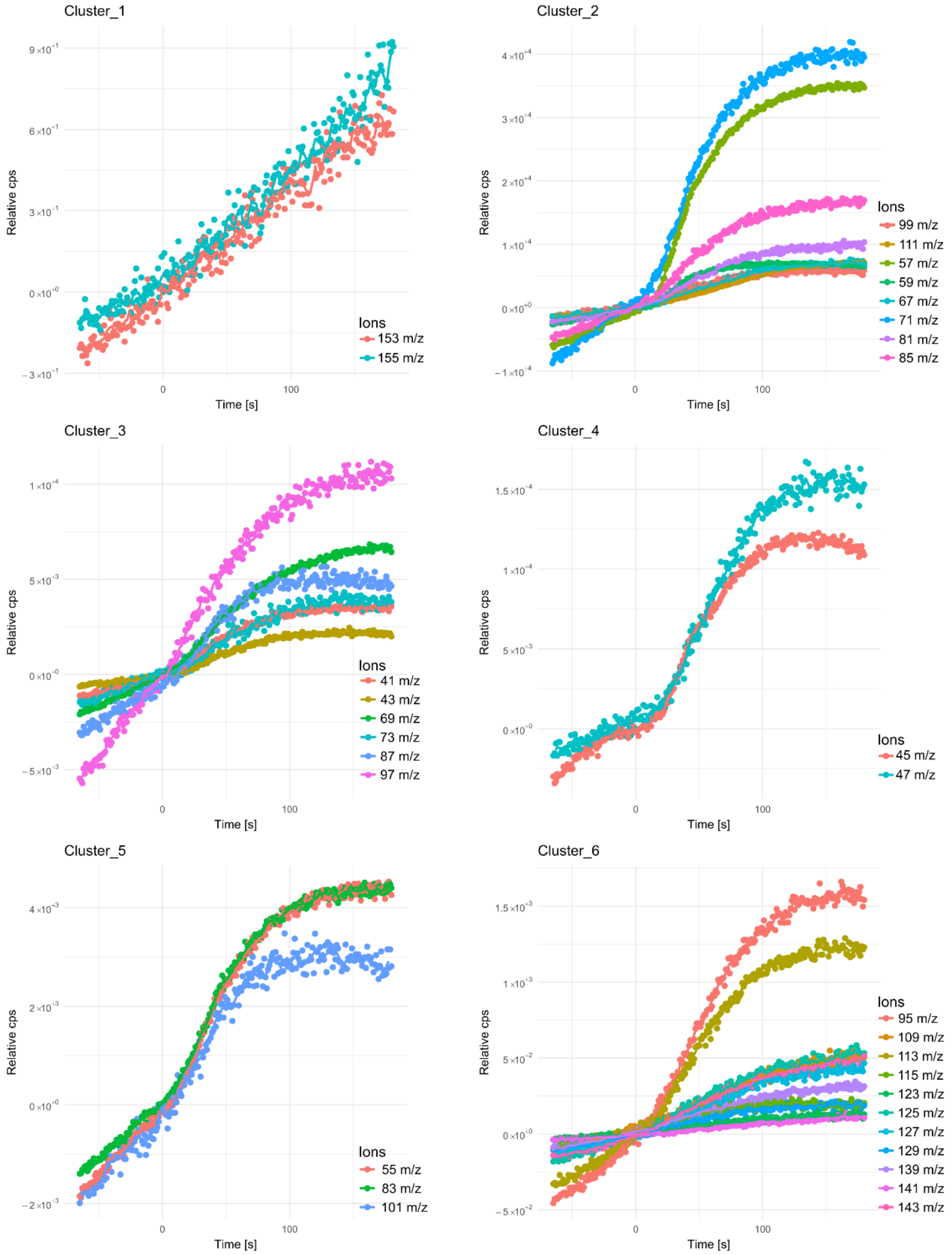

Figure S3. The emission profiles of individual VOCs (grouped in clusters) monitored during deep frying of tempeh in rapeseed oil; frying at 180 °C, third use of oil; y-axis – relative counts per second, where zero is the start of frying; x-axis – frying time before frying (below zero) and after placing tempeh; the line is the result of smoothing with moving average with step = 5

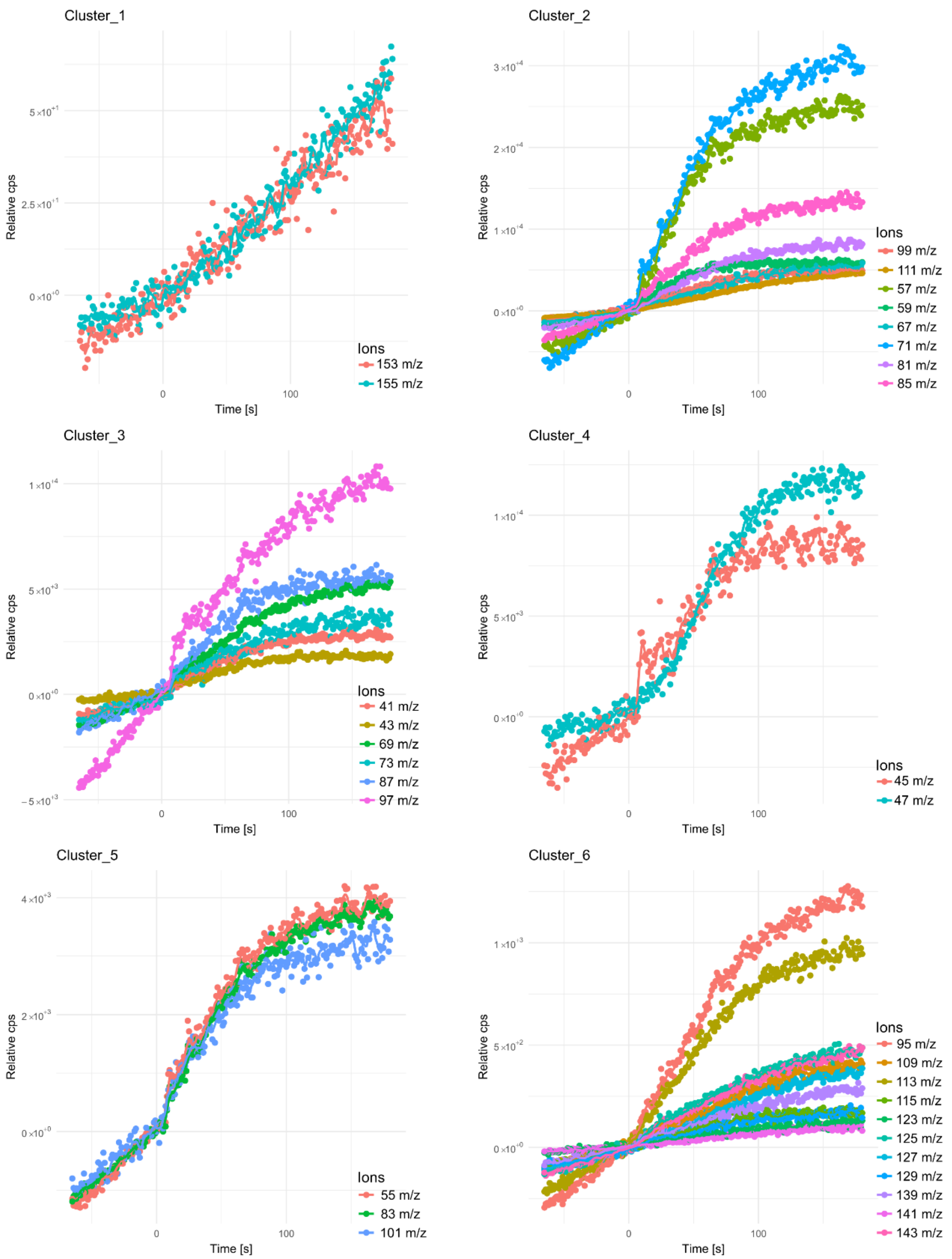

Figure S4. The emission profiles of individual VOCs (grouped in clusters) monitored during deep frying of tempeh in rapeseed oil; frying at 180 °C, fourth use of oil; y-axis – relative counts per second, where zero is the start of frying; x-axis – frying time before frying (below zero) and after placing tempeh; the line is the result of smoothing with moving average with step = 5

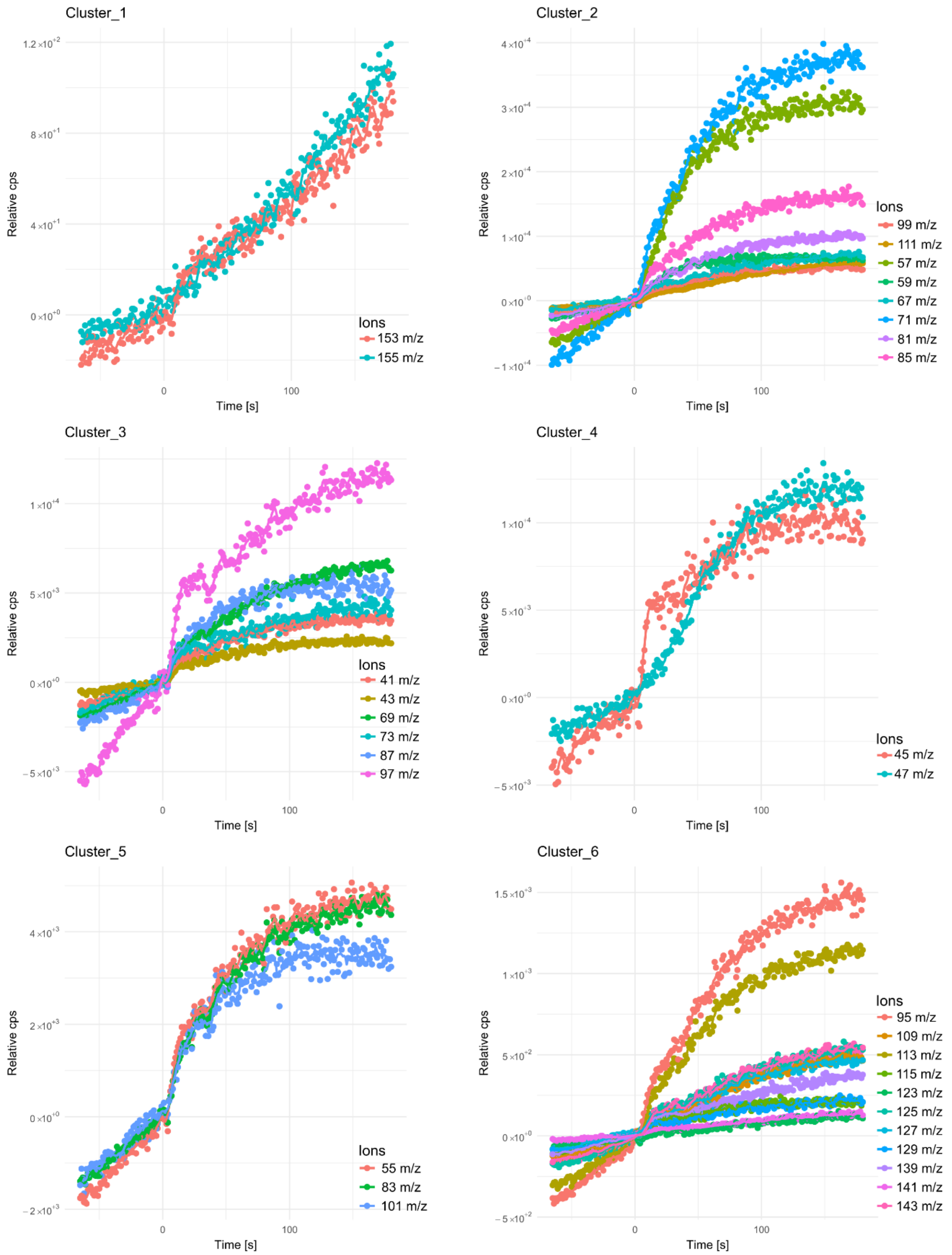

Figure S5. The emission profiles of individual VOCs (grouped in clusters) monitored during deep frying of tempeh in rapeseed oil; frying at 180 °C, fifth use of oil; y-axis – relative counts per second, where zero is the start of frying; x-axis – frying time before frying (below zero) and after placing tempeh; the line is the result of smoothing with moving average with step = 5

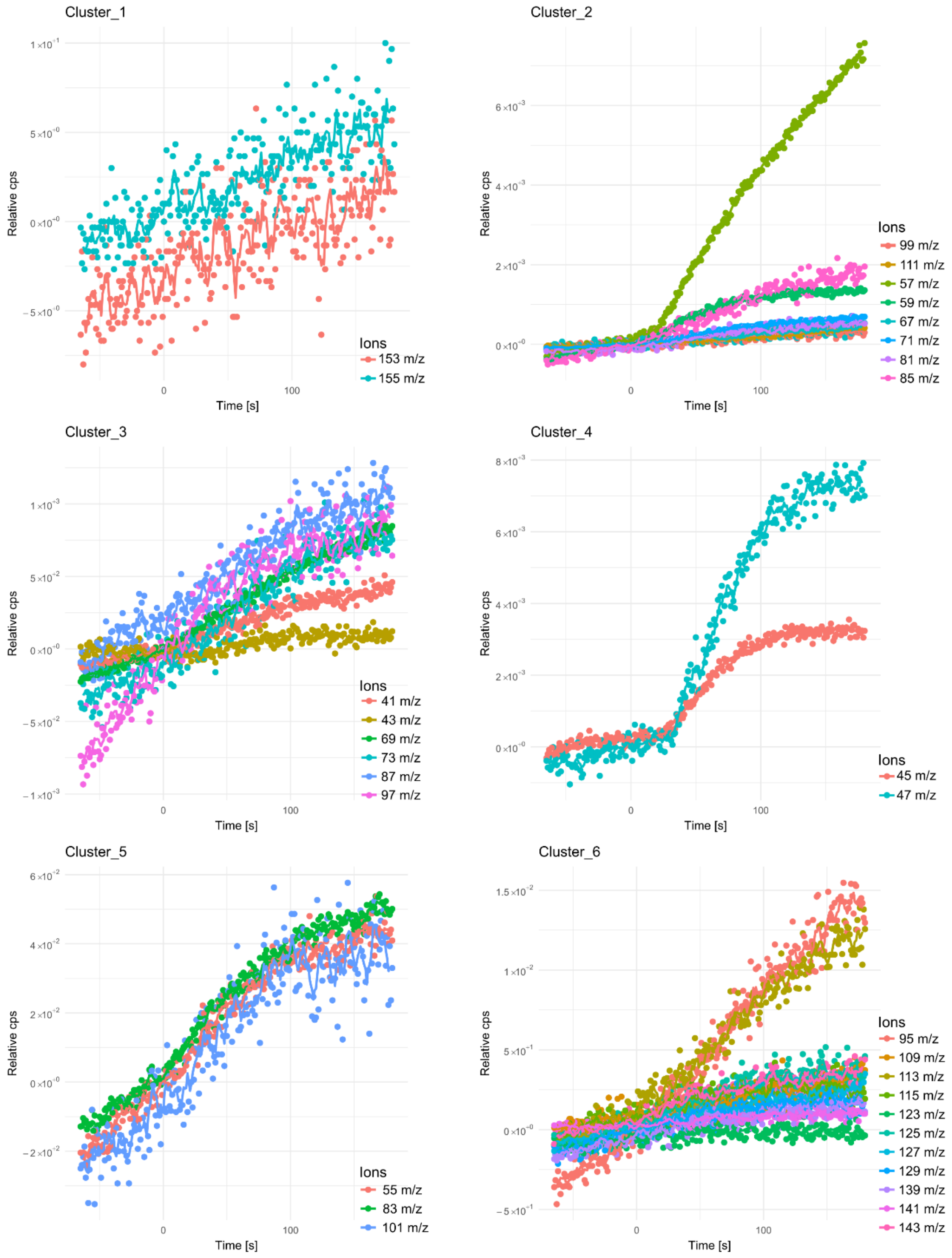

Figure S6. The emission profiles of individual VOCs (grouped in clusters) monitored during deep frying of tempeh in rapeseed oil; frying at 160 °C, fresh oil; y-axis – relative counts per second, where zero is the start of frying; x-axis – frying time before frying (below zero) and after placing tempeh; the line is the result of smoothing with moving average with step = 5

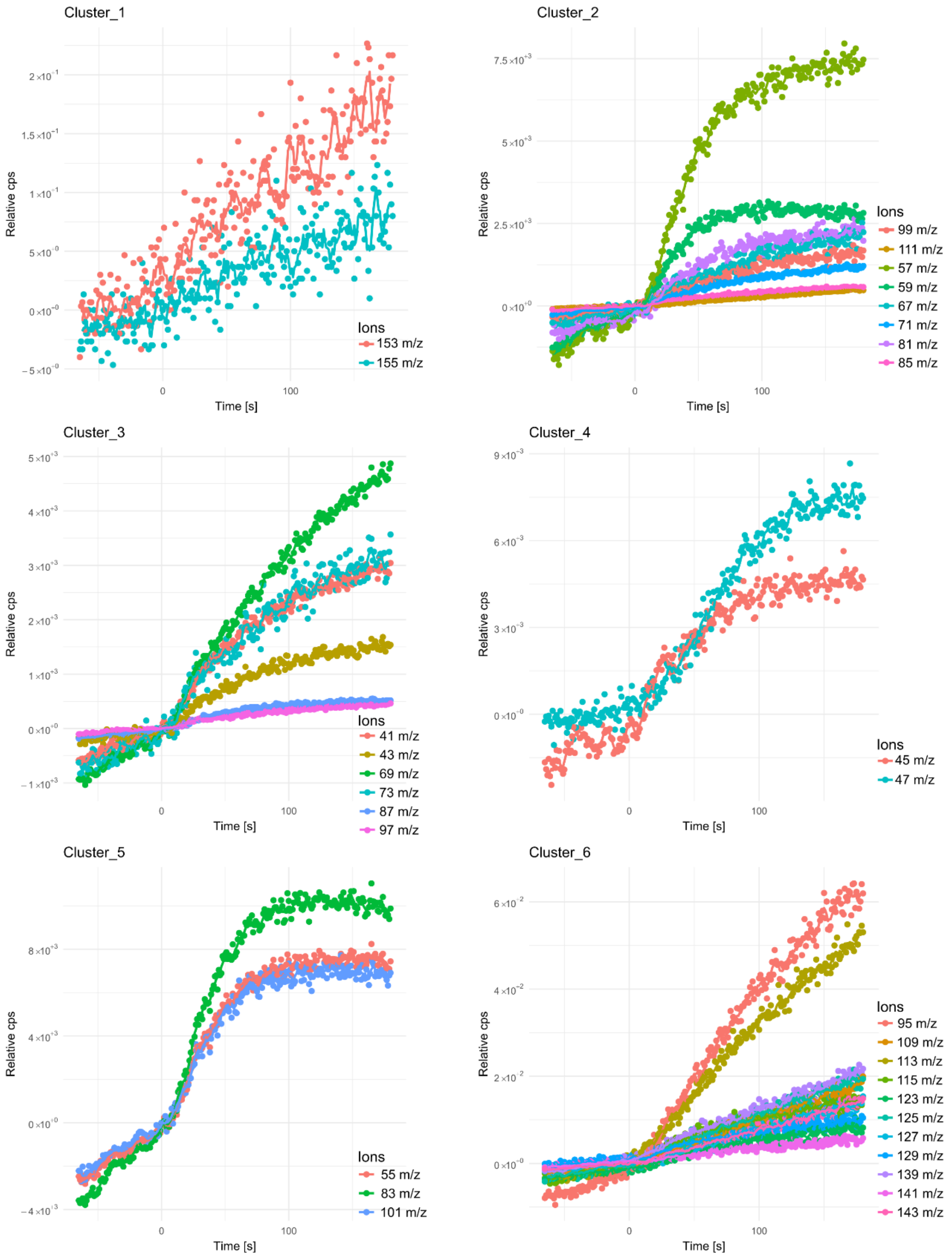

Figure S7. The emission profiles of individual VOCs (grouped in clusters) monitored during deep frying of tempeh in palm oil; frying at 180 °C, fresh oil; y-axis – relative counts per second, where zero is the start of frying; x-axis – frying time before frying (below zero) and after placing tempeh; the line is the result of smoothing with moving average with step = 5

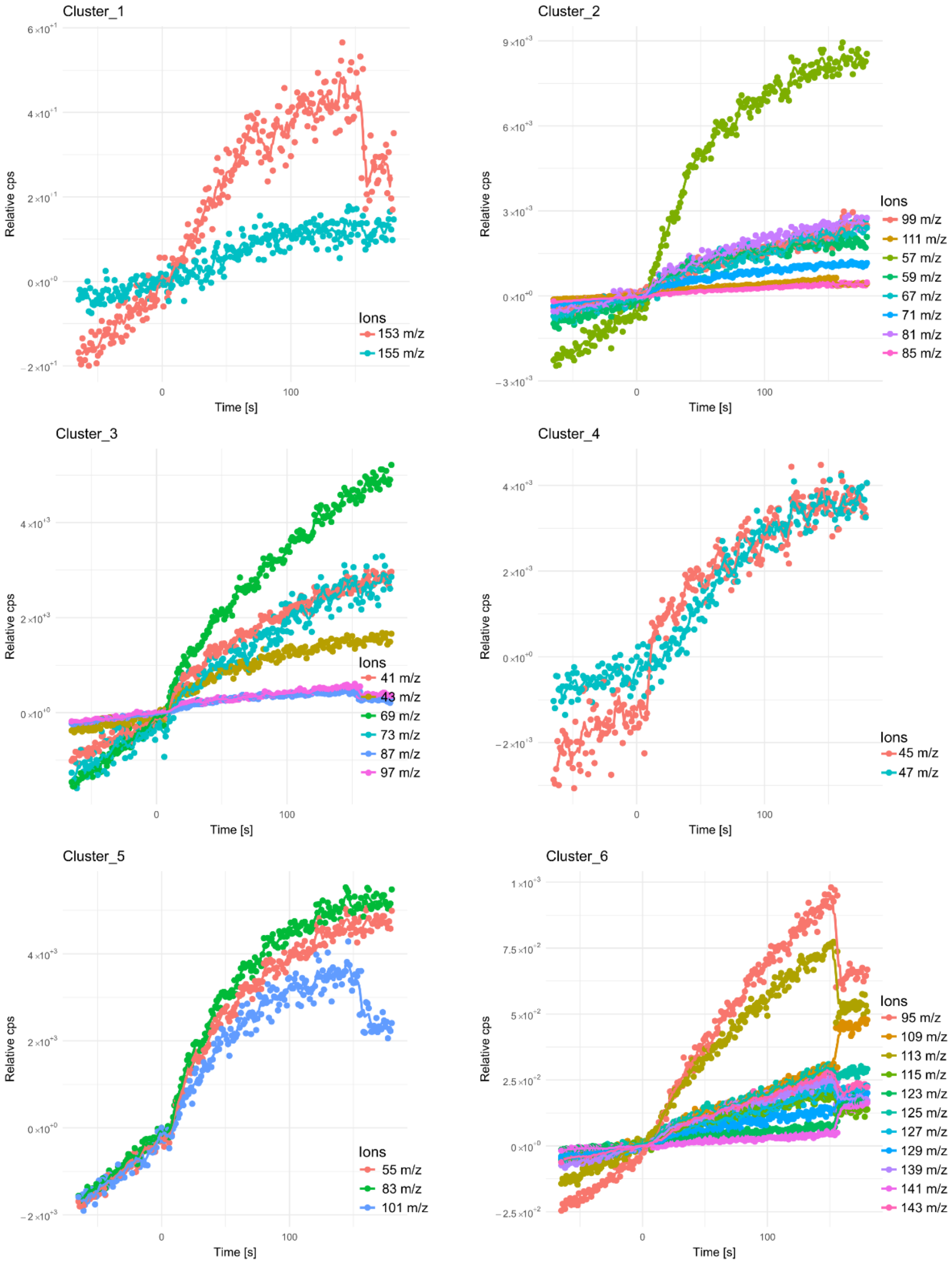

Figure S8. The emission profiles of individual VOCs (grouped in clusters) monitored during deep frying of tempeh in palm oil; frying at 180 °C, second use of oil; y-axis – relative counts per second, where zero is the start of frying; x-axis – frying time before frying (below zero) and after placing tempeh; the line is the result of smoothing with moving average with step = 5; unexpected signal drop/rise for some ions is due to the m/z axis calibration issues

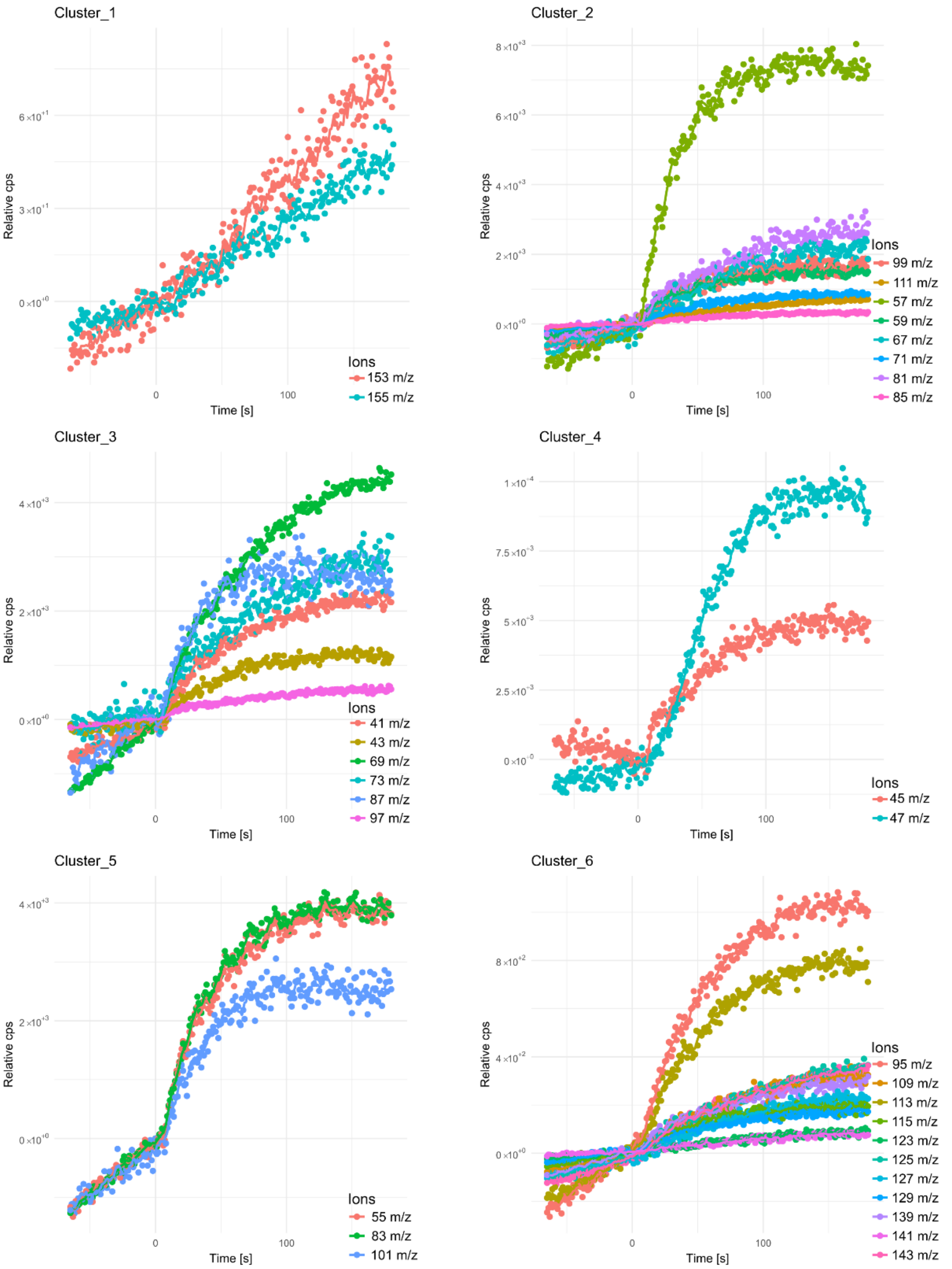

Figure S9. The emission profiles of individual VOCs (grouped in clusters) monitored during deep frying of tempeh in palm oil; frying at 180 °C, third use of oil; y-axis – relative counts per second, where zero is the start of frying; x-axis – frying time before frying (below zero) and after placing tempeh; the line is the result of smoothing with moving average with step = 5

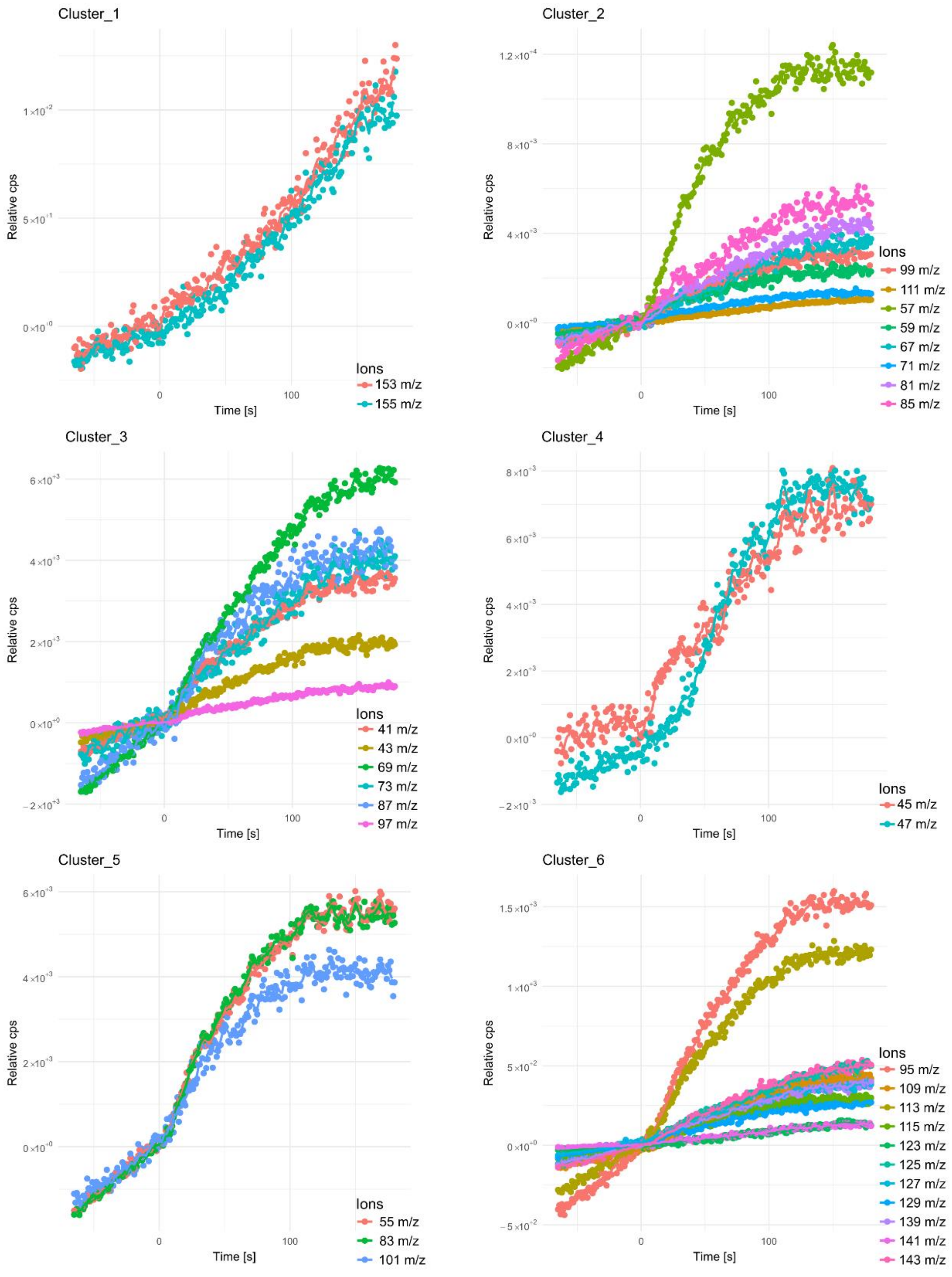

Figure S10. The emission profiles of individual VOCs (grouped in clusters) monitored during deep frying of tempeh in palm oil; frying at 180 °C, fourth use of oil; y-axis – relative counts per second, where zero is the start of frying; x-axis – frying time before frying (below zero) and after placing tempeh; the line is the result of smoothing with moving average with step = 5

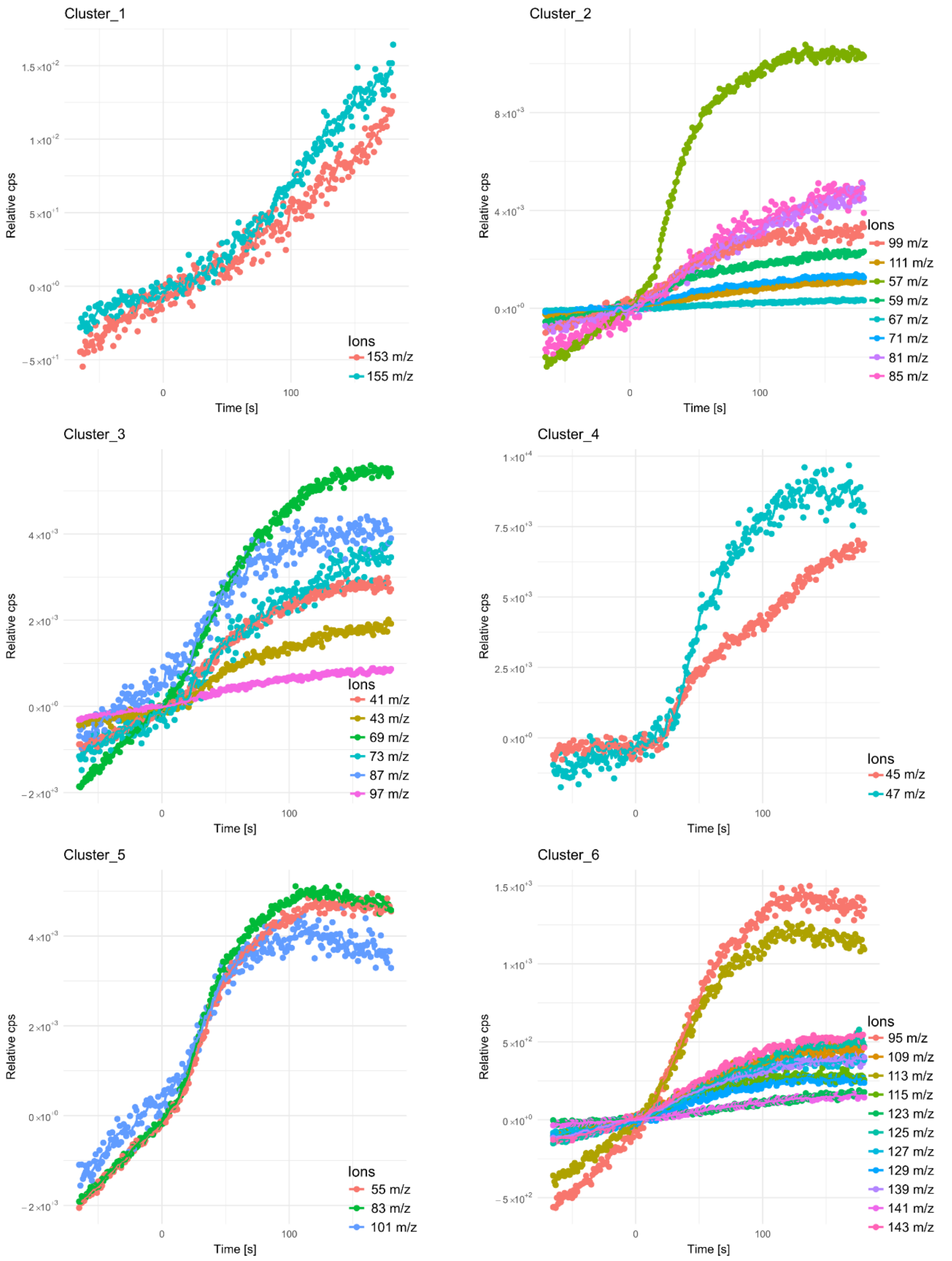

Figure S11. The emission profiles of individual VOCs (grouped in clusters) monitored during deep frying of tempeh in palm oil; frying at 180 °C, fifth use of oil; y-axis – relative counts per second, where zero is the start of frying; x-axis – frying time before frying (below zero) and after placing tempeh; the line is the result of smoothing with moving average with step = 5

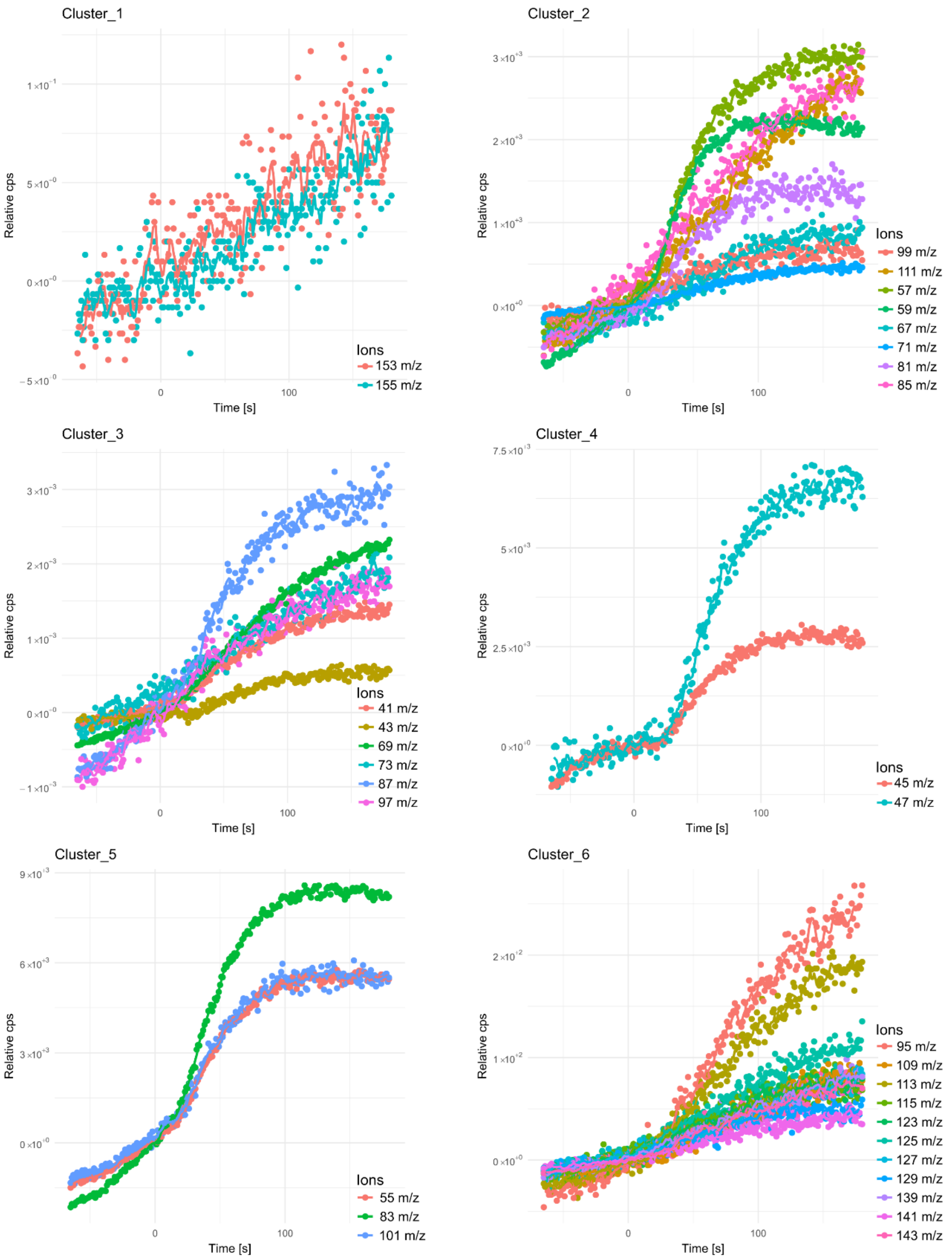

Figure S12. The emission profiles of individual VOCs (grouped in clusters) monitored during deep frying of tempeh in palm oil; frying at 160 °C, fresh oil; y-axis – relative counts per second, where zero is the start of frying; x-axis – frying time before frying (below zero) and after placing tempeh; the line is the result of smoothing with moving average with step = 5
